# Renal tumors with different histological types occurring in the same kidney: A case report

**DOI:** 10.1016/j.eucr.2025.102991

**Published:** 2025-02-19

**Authors:** Maeda Akihiro, Kazuma Udo, Masahiro Ito, Maki Kawasaki, Hiroaki Kakinoki, Shohei Tobu, Mitsuru Noguchi

**Affiliations:** a Department of Urology, Faculty of Medicine, Saga University, Japan; b Department of Urology, Saga University Faculty of Medicine, Saga, Japan

**Keywords:** The same kidney, Simultaneous occurrence, Different histological types, Renal tumor

## Abstract

We report on one rare clinical case where renal tumors of different histological types occurred simultaneously. An 84-year-old male with two right renal tumors underwent radical nephrectomy. Pathology revealed chromophobe renal cell carcinoma (ChRCC) and clear cell renal cell carcinoma (ccRCC). He had a 3-year recurrence-free survival without renal function decline.

## 1 Introduction

Simultaneous renal tumors in the same kidney are rare and most commonly associated with hereditary syndromes, such as Von Hippel–Lindau disease and Birt–Hogg–Dubé syndrome.^1^^,^^2^ However, reports of renal tumors with different histological types occurring simultaneously in the same kidney are extremely uncommon.

We herein report one case of synchronous renal tumors with distinct histological subtypes arising in the same kidney in patients without hereditary syndromes, along with a literature review.

## 2 Case presentation

Between January 2013 and December 2023, 327 patients underwent nephrectomy or partial nephrectomy for renal tumors at our hospital. Among these, 2 cases (0.6 %) involved synchronous renal tumors with different histological subtypes in the same kidney. One of the cases involved clear cell RCC (ccRCC) and angiomyolipoma (AML). The other case consisted of both renal tumors being malignant. Here, we provide a detailed description of this case.

**Fig. 1:**
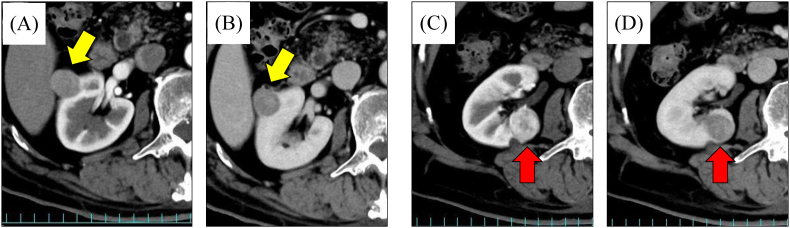
Enhanced CT showed a tumor measuring 20 × 19 mm on the ventral side of the right kidney (the yellow arrow indicated). The tumor showed no enhancement during the early (A) or the late (B) phases. Enhanced CT showed a tumor measuring 21 × 20 mm on the renal hilum of the right kidney (the red arrow indicated). The tumor showed strong contrast effect at the early phase (C), with disappearance of this effect at the late phase (D).

An 84-year-old male with full activities of daily living (ADL) was referred to our hospital after plain computed tomography (CT) for a preoperative evaluation of spinal canal stenosis revealed a right renal tumor. Contrast-enhanced CT identified two distinct renal tumors in the right kidney with differing characteristics (Fig. 1).

One tumor, located on the ventrolateral side, measured 20 × 19 mm and showed poor enhancement. Hypovascular renal cell carcinoma, such as chromophobe renal cell carcinoma (ChRCC) or papillary RCC, was suspected (cT1aN0M0, R.E.N.A.L. score^3^ 5a). The other tumor, located medially near the renal hilum, measured 21 × 20 mm, and exhibited an enhancement pattern suggestive of ccRCC (cT1aN0M0, R.E.N.A.L. score 8p).

**Fig. 2:**
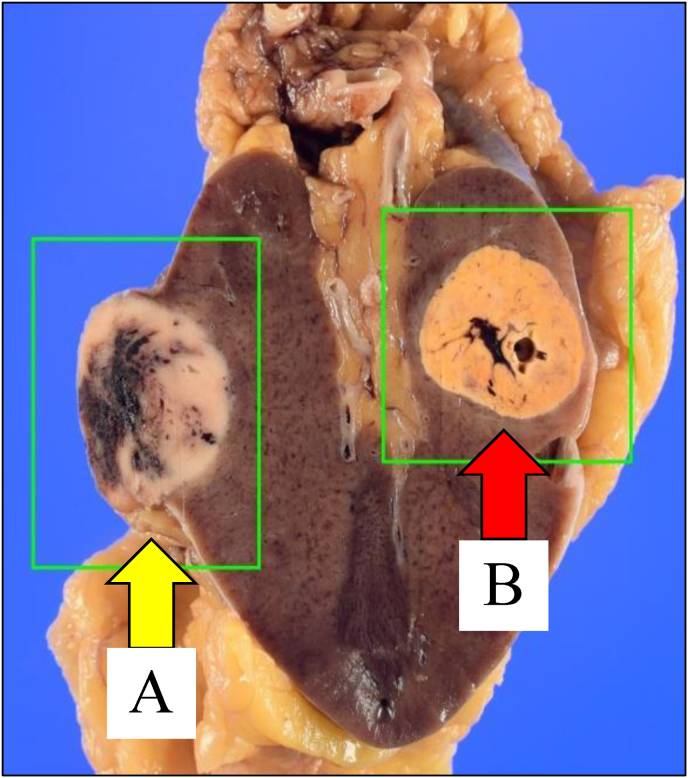
Macroscopic image of the right kidney. (A)A tumor measuring 22 × 20 mm on the ventral side. (B)A tumor measuring 20 × 18 mm on the renal hilum.

**Fig. 3:**
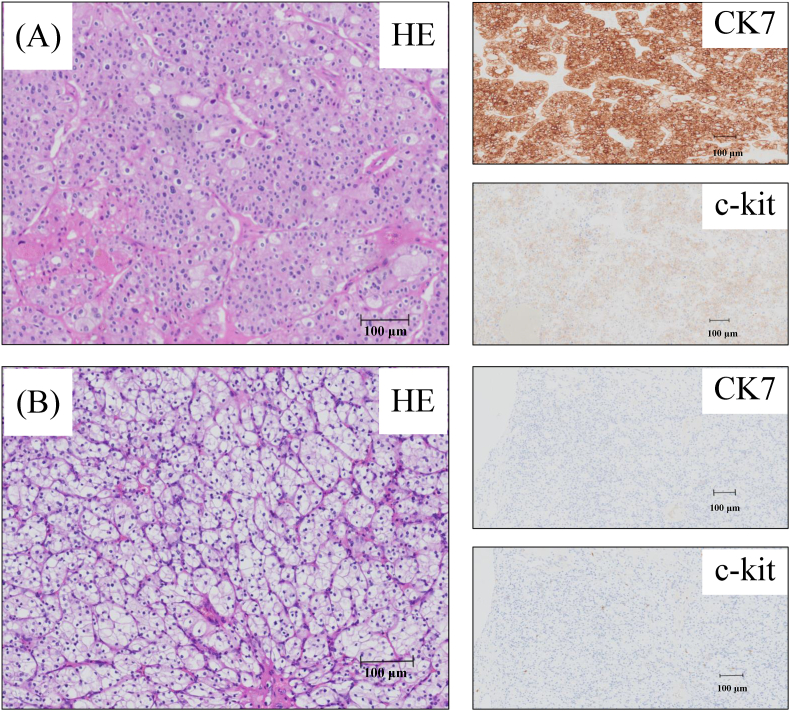
(A)Showed the pathological findings of the tumor on the ventral side. Hematoxylin and Eosin (HE) staining exhibited a mixture of large polygonal cells with abundant flocculent cytoplasm and small cells with eosinophilic cytoplasm. Immunohistochemical (IHC) staining showed positive staining to CK7 and c-kit, consistent with a diagnosis of ChRCC. (B) Showed the pathological findings of the tumor on the renal hilum. HE staining exhibited compact nests and sheets of cells with clear cytoplasm and distinct membrane. IHC staining showed negative staining to CK7 and c-kit, consistent with a diagnosis of ccRCC.

Considering curative intent, retroperitoneoscopic right nephrectomy was performed without intraoperative or postoperative complications. A pathological examination revealed the following findings. The ventral tumor, on hematoxylin and eosin (HE) staining, exhibited a mixed pattern of large polygonal cells with abundant flocculent cytoplasm and small cells with eosinophilic cytoplasm. Immunohistochemical (IHC) staining was positive for CK7 and c-kit, consistent with a diagnosis of ChRCC (pT1a). The dorsal tumor, on HE staining, exhibited compact nests and sheets of cells with clear cytoplasm and distinct membranes. IHC staining was negative for CK7 and c-kit, consistent with a diagnosis of ccRCC (pT1a) (Fig. 2, Fig. 3).

Postoperatively, regular imaging and blood tests showed no signs of recurrence or metastasis over the three-year follow-up period. Renal function monitoring showed a decline in the estimated glomerular filtration rate (eGFR) from 85 ml/min/1.73 m^2^ preoperatively (CKD stage G2) to 46 ml/min/1.73 m^2^ postoperatively (CKD stage G3a), reflecting a 45 % decrease. However, no symptoms of renal failure were observed.

## 3 Discussion

The proportion of cases with multiple renal tumors, where each tumor is histopathologically different, has been reported to range from 6 % to 30 %.^4^ Furthermore, the occurrence of tumors with different histological subtypes simultaneously in the same kidney is rare. In our department's experience, this was observed in only 0.6 % of the 327 patients who underwent nephrectomy or partial nephrectomy. Dimarco et al. reported that among, 2373 cases of nephrectomy performed for RCC, simultaneous occurrence of multiple tumors in the same kidney was observed in 130 cases (5.4 %). Among these, cases such as Case 1, where ChRCC and ccRCC occurred simultaneously, were extremely rare, with only 2 instances reported (0.08 % of the total).^5^ Recent genetic analyses have suggested that ChRCC originates from distal nephron cell of origin, while ccRCC arises from proximal nephron cell of origin.^6^ Since their sites of origin differ, the simultaneous occurrence of these subtypes is considered extremely rare.

The coexistence of AML and RCC is also rare. The simultaneous occurrence of renal tumors, including both AML and RCC, was reported in 18 (0.8 %) of 2160 nephrectomy cases.^7^ In addition, 33 cases of simultaneous occurrence of AML and RCC in the same kidney have been reported, of which 17 were in patients with tuberous sclerosis, while 16 (48 %) were in non-tuberous sclerosis patients, such as in Case 2.^7^, ^8^, ^9^, ^10^

Regarding surgical treatment for synchronous renal tumors in the same kidney, traditionally, radical nephrectomy (RN) has been the treatment of choice for synchronous renal tumors in the same kidney. However, recent reports indicate that partial nephrectomy (PN) is more commonly performed in such cases, aided by the widespread use of robot-assisted surgery. PN has been shown to provide equivalent cancer-specific survival rates and better postoperative renal function preservation than RN.^11^ According to Mano et al., among 123 patients with synchronous renal tumors in the same kidney, 78 underwent PN, and 45 underwent RN. The 5-year recurrence-free survival rates were 98 % and 85 % in the PN and RN groups, respectively. In addition, the proportion of patients who experienced renal function deterioration over 5 years was 26 % in the PN group compared with 45 % in the RN group.^12^ These findings suggest that PN may offer a lower recurrence rate and better renal function preservation than RN. Therefore, PN can be considered a treatment option for patients with synchronous renal tumors in the same kidney, provided that careful patient selection is conducted.

## 4 Conclusion

We herein report one case of synchronous renal tumors with different histological subtypes in the same kidney, which is a rare clinical occurrence.

## CRediT authorship contribution statement

**Maeda Akihiro:** Writing – review & editing, Writing – original draft, Resources, Methodology, Conceptualization. **Kazuma Udo:** Writing – review & editing, Project administration, Investigation, Conceptualization. **Masahiro Ito:** Visualization, Investigation, Formal analysis. **Maki Kawasaki:** Validation. **Hiroaki Kakinoki:** Validation, Investigation. **Shohei Tobu:** Validation, Investigation. **Mitsuru Noguchi:** Supervision.

## Informed consent

Not applicable.

## Funding source

None.

## Declaration of competing interest

The authors declare no conflicts of interest.
